# Bond strength of selected composite resin-cements to zirconium-oxide ceramic

**DOI:** 10.4317/medoral.18243

**Published:** 2012-08-28

**Authors:** Juan L. Román-Rodríguez, Antonio Fons-Font, Vicente Amigó-Borrás, María Granell-Ruiz, David Busquets-Mataix, Rubén A. Panadero, Maria F. Solá-Ruiz

**Affiliations:** 1 Occlusion and Prosthodontic Teaching Unit, Department of Stomatology, University of Valencia, Spain; 2 Institute of Materials Technology, Polytechnic University, Valencia, Spain; 3 ….

## Abstract

Objectives: The aim of this study was to evaluate bond strengths of zirconium-oxide (zirconia) ceramic and a selection of different composite resin cements. Study Design: 130 Lava TM cylinders were fabricated. The cylinders were sandblasted with 80 µm aluminium oxide or silica coated with CoJet Sand. Silane, and bonding agent and/or Clearfil Ceramic Primer were applied. One hundred thirty composite cement cylinders, comprising two dual-polymerizing (Variolink II and Panavia F) and two autopolymerizing (Rely X and Multilink) resins were bonded to the ceramic samples. A shear test was conducted, followed by an optical microscopy study to identify the location and type of failure, an electron microscopy study (SEM and TEM) and statistical analysis using the Kruskal-Wallis test for more than two independent samples and Mann-Whitney for two independent samples. Given the large number of combinations, Bonferroni correction was applied (α=0.001). Results: Dual-polymerizing cements provided better adhesion values (11.7 MPa) than the autopolymerizing (7.47 MPa) (p-value M-W<0.001). The worst techniques were Lava TM + sandblasting + Silane + Rely X; Lava TM + sandblasting + Silane + Multilink and Lava TM + CoJet + silane + Multilink. Adhesive failure (separation of cement and ceramic) was produced at a lesser force than cohesive failure (fracture of cement) (p-value M-W<0.001). Electron microscopy confirmed that the surface treatments modified the zirconium-oxide ceramic, creating a more rough and retentive surface, thus providing an improved micromechanical interlocking between the cement and the ceramic.

** Key words:**Shear bond strength, silica coating, surface treatment, zirconia ceramics, phosphate monomer.

## Introduction

Continued development in ceramic materials has allowed the restorative dentist to extend their indications for use. These materials can now be used for single or multiple bonded ceramic restorations, dental implants and implant abutments among others (1).

Ceramics are classified according to their base, silicate or oxide, the latter being either aluminum or zirconium oxide (2).

Resin cements are currently indicated for cementing ceramic restorations (3-6). These restorations require an internal treatment according to their ceramic composition in order to optimize the ceramic-cement bond (1,7). Resin cements bond by friction and by adhesion, understanding adhesion to be the close bond formed between two materials of a different chemical nature (2,4,8).

This adhesive bonding is based on two factors, one micromechanical and the other chemical. The micromechanical component refers to the interlocking of the resin cement with the previously treated roughened ceramic surface. Silicate ceramics are roughened by hydrofluoric acid etching, whereas the oxide ceramics, lacking silica, are sandblasted or silica coated (2,9).

The chemical component is based on a chemical bond between the two materials produced by products such as silane or 10-methacryloyloxydecyl dihydrogen phosphate (MDP). Silane (3-methacryloyxpropytrimethoxy silane) possesses two characteristics that facilitate the adhesion between the inorganic substrate (ceramic) and the organic polymers (adhesive and/or resin cement). Firstly, it increases the wettability of the porcelain, facilitating interlocking. Secondly, it is a bifunctional molecule with silane groups at one end (ionic bonding with the silicon in the ceramic) and methacrylate groups at the other, which can react and bond with the methacrylate groups in the resin (covalent bonding) (10). Silanization of the silicate ceramics improves adhesion with the resin, although some authors disagree as to its efficacy in oxide ceramics (4,5) Currently, silane may be applied to the oxide ceramics alone or used in combination with MDP. MDP is a long organic hydrophobic chain molecule with two ends. One end has vinyl groups that react with the monomers of the resin cement when polymerized. At the other end, hydrophilic phosphate ester groups bond strongly with metal oxides such as alumina (Al2O3) and zirconium (ZrO2), or where necessary, with calcium hydroxyapatite (10).

Cement bonding of oxide ceramics has been extensively studied (2,7-9,11-16); nevertheless, a reliability similar to that provided by silicate ceramics and resin cements has yet to be attained. The present study evaluates different methods of zirconium-oxide surface treatments in combination with different chemical agents. Obtaining a good cement-ceramic bond is essential, as it increases the strength of the restoration, decreases the possibility of tooth fracture, improves the marginal adaptation of the restoration, preventing secondary caries and changes in cement color, and decreases the likelihood of debonding in cases of short or tapered abutments (1,17).

The aim of this study was to analyze the bond strength between zirconia ceramic and composite resin cements. An optical and electron microscopy study was also carried out in order to understand the effects of the different ceramic surface treatments performed.

## Material and Methods

One hundred thirty zirconium-oxide ceramic cylinders were fabricated (Ø 5 mm x length 7 mm) (Y-TZP-A: Y-TZP-A: yttrium oxide-stabilized tetragonal zirconio polycrystals doped with alumina) (Lava TM System Frame®, 3M ESPE, Seefeld, Germany) and retained in copper cylinders filled with plaster.

Similarly, 130 composite cement cylinders (Ø 5 mm x 7 mm length) were made, comprising 4 types of cement; two dual-polymerizing (Variolink II®, Ivoclar Vivadent, Schanne, Liechtenstein, and Panavia F ®, Kuraray, Osaka, Japan) and two autopolymerizing (Rely X ®, 3M ESPE, and Multilink ®, Ivoclar Vivadent).

Forty ceramic cylinders were sandblasted with 80 micron aluminum-oxide particles, 3 bars for 10 seconds; and ninety ceramic cylinders blasting with 30 micron aluminum-oxide silica-coated particles (CoJet Sand ®, 3M ESPE), 2 - 3 bars, 10-15 seconds. Silane, a bonding agent, or a bonding agent possessing both MDP and silane (Clearfil Ceramic Primer ®, Kuraray) were applied depending on the test group.

The composite cement cylinders were bonded to the ceramic cylinders, establishing 13 test groups (n=10). The samples were kept for 24 hours at 37 ºC in a JP Selecta model 210 oven (Abrera, Spain).

 Table 1 shows the composition of the 13 test groups.

**Table 1:**
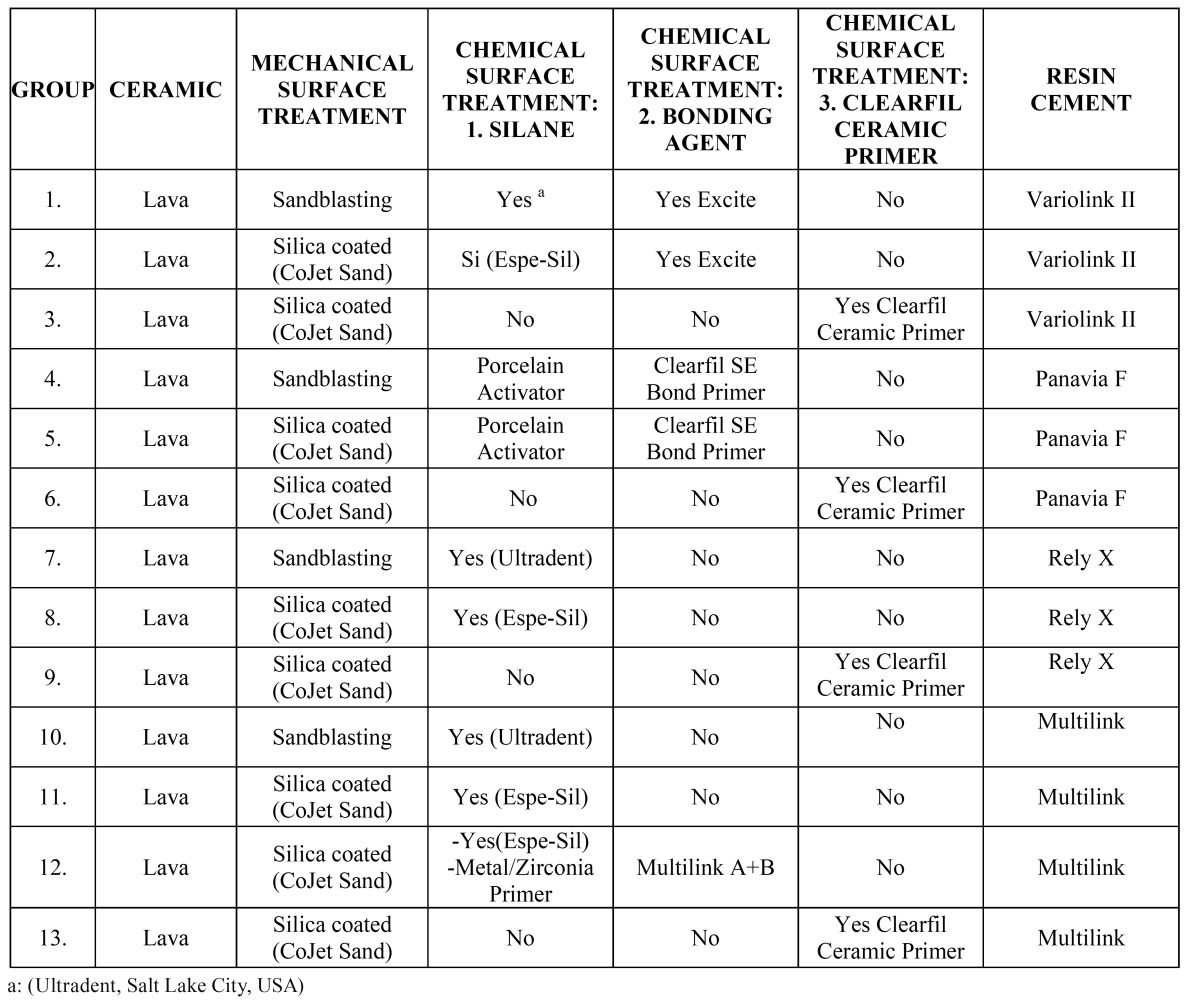
Description of the 13 study groups and the materials used.

**Figure 1:**
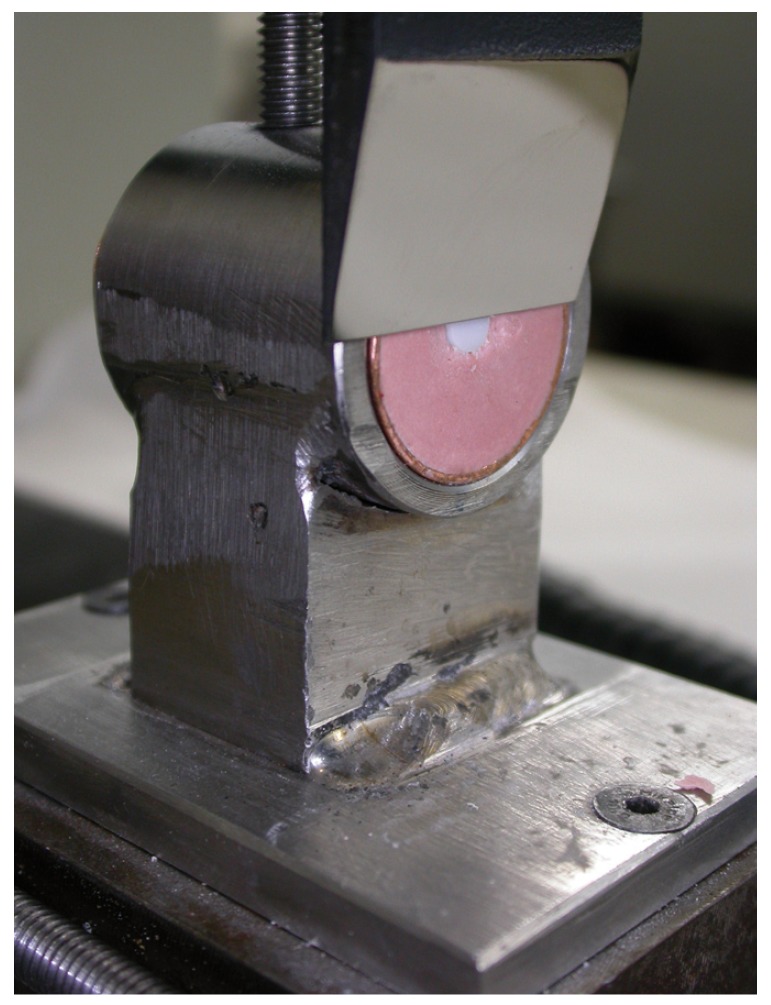
Completed shear test, showing the ceramic cylinder with the Instron blade having removed the composite cement cylinder.

A shear test was performed using an Instron 4202 with a load of 1 kN and a crosshead speed of 0.5 mm/min (Fig. 1). After the test the ceramic cylinder was examined with an optical microscope (Nikon® SMZ-10a and Nikon® Microfot FX, Tokyo, Japan) to locate the point of failure, whether in the ceramic cylinder (cohesive failure C1), in the composite material (cohesive failure C2) or in the composite-ceramic bond (adhesive failure A).

Four additional ceramic cylinders were fabricated for examination by scanning electron microscopy (SEM) (JEOL JSM 6300 with crystal microanalysis Oxford Instruments Ltd, Tokyo, Japan) and transmission electron microscopy (TEM) (Philips CM-10 equipped with CCD for image capture, Amsterdam, Netherlands), and were not subjected to shear testing.

TEM images are based on passing an electron beam through a fine lamina (1-5 nm thick) taken from the zirconia sample. TEM provides very precise measurements due to the magnification used and because of the two-dimensional nature of the wafer-thin sample.

Finally, all data were statistically analyzed using Kruskal-Wallis test and Mann-Whitney test with Bonferroni correction applied (α = 0.001).

## Results

-Statistical analysis

Significant differences were found between the adhesion values of the different groups tested (p-value K-W<0.001). Three com-binations of groups were distinguished according to the adhesion values obtained ( Table 2, Table 3, Table 4). The combination of groups with the highest values showed statistically significant differences from those of the lowest, whereas the intermediate groups showed no significant differences between either the highest or lowest groups ( Table 2, Table 3). It was observed that group 4 (Lava + sandblasting + Clearfil SE Bond Primer + Porcelain Activator + Panavia F = 14.90 ± 5.01) achieved the highest values in the study, followed by group 3 (Lava + CoJet Sand + Clearfil Ceramic Primer + Variolink II = 13.44 ± 3.53), although without significant differences with groups 5,2,1,8 and13 ( Table 3).

**Table 2:**
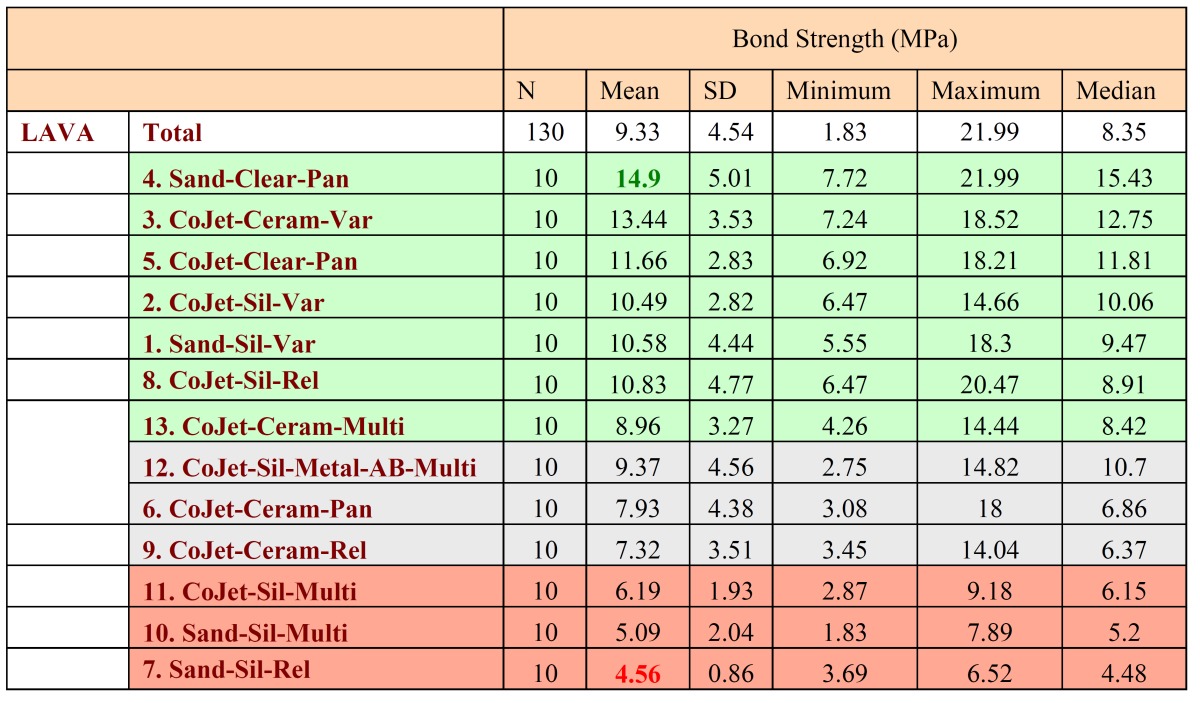
Shear test results and descriptive statistics.

**Table 3:**
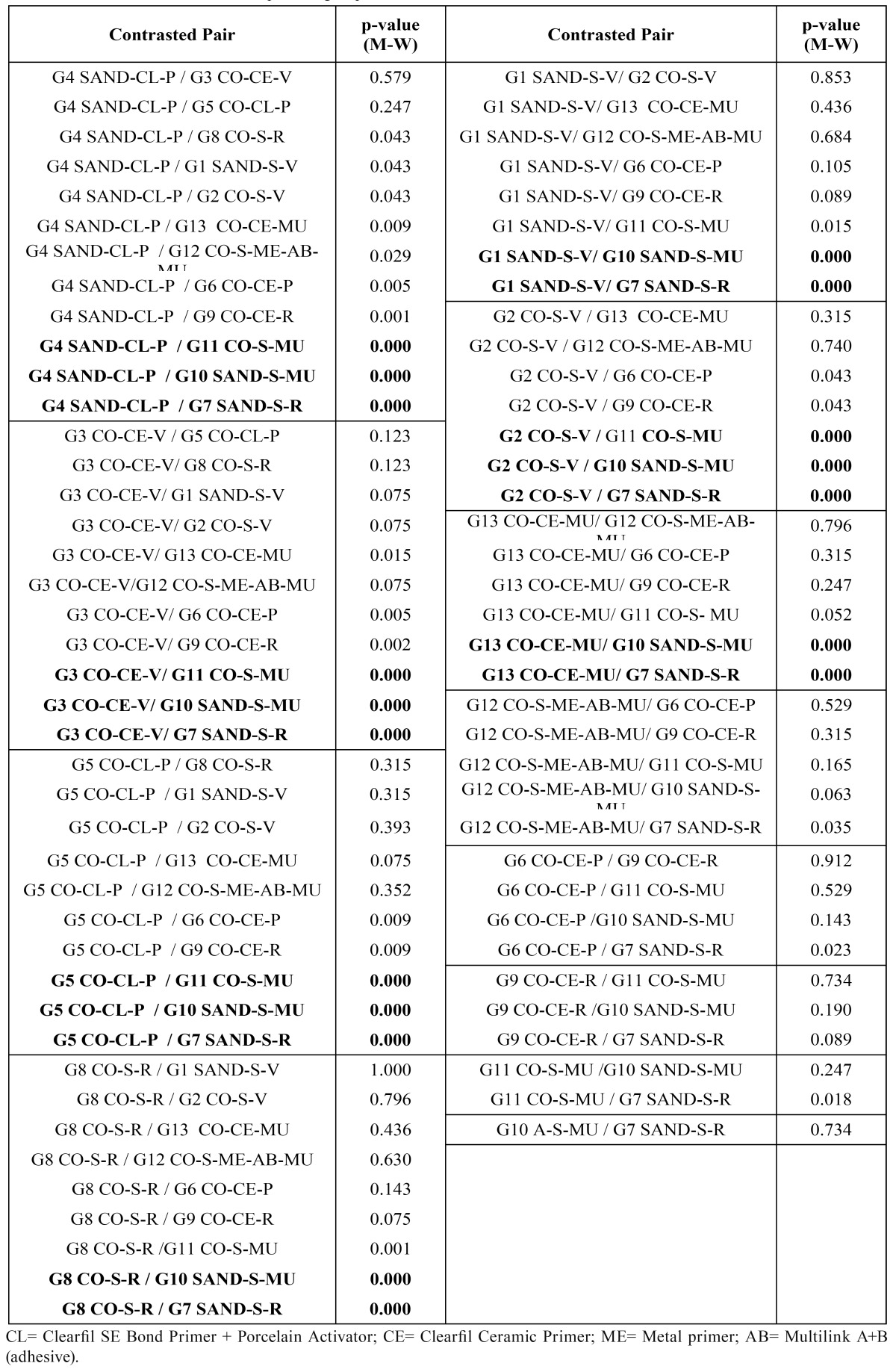
Statistical contrasts between pairs of groups.

**Table 4:**
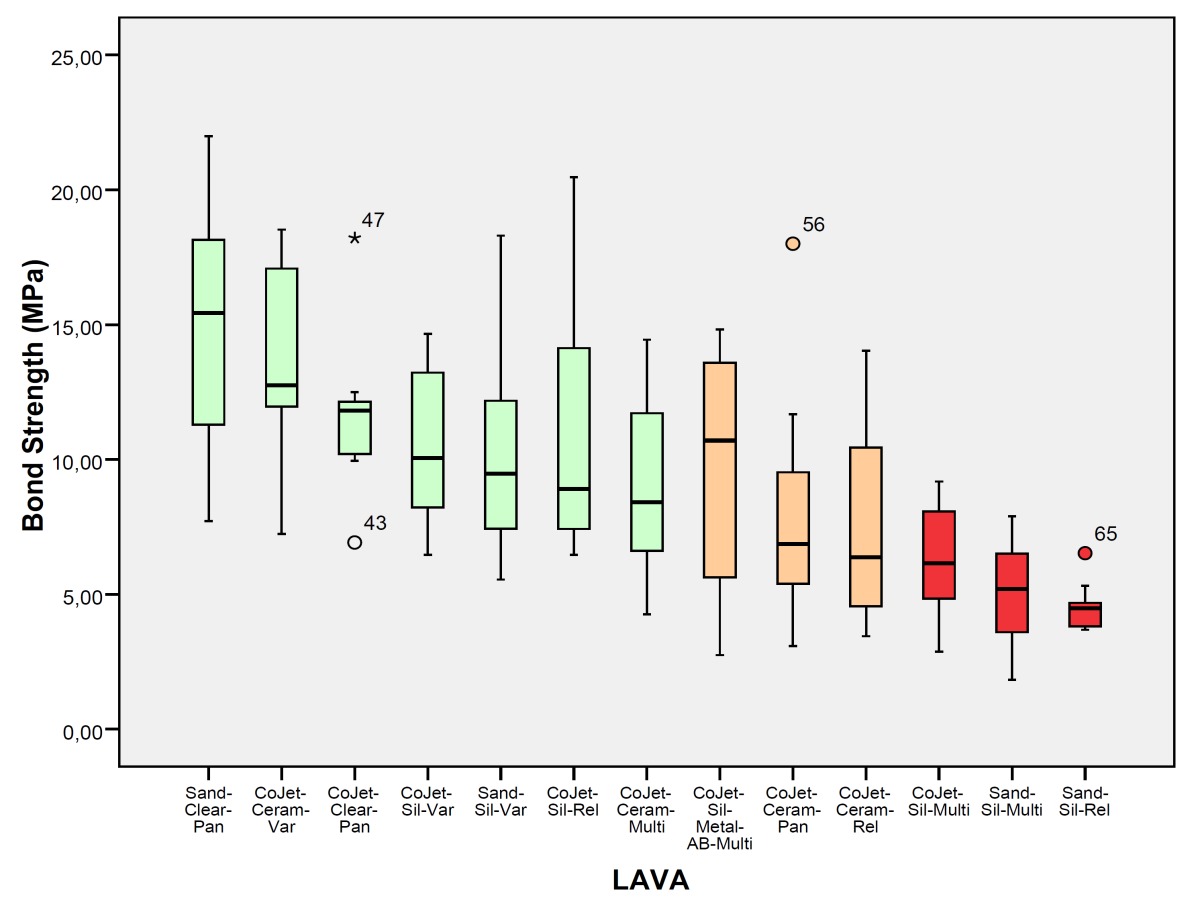
Box-Plot of the shear test carried out on the different test groups.

The worst values corresponded to groups 7 (Lava + sandblasting + silane + Rely X = 4.56 ± 0.86), 10 (Lava + sandblasting + silane + Multilink = 5.09 ± 2.04) and 11 (Lava + CoJet Sand + silane + Multilink = 6.19 ± 1.93) ( Table 2).

The dual-polymerizing cements presented generally better bond strengths than the autopolymerizing (p-value M-W<0.001). It is not possible to speak in absolute terms of a better cement (Panavia vs Variolink II: p-value M-W 0.734) (Rely X vs Multilink: p-value M-W 0.849), or a better surface treatment (p-value M-W 0.089), since each technique is a combination of up to four interactive elements. The autopolymerizing cements provide better results when silica coated than with sandblasting (p-value M-W <0.001).

-Optical Microscopy

No cohesive failures were observed in the ceramic (C1), whereas 82 adhesive failures (A) and 48 cohesive cement failures (C2) were found. Comparison with the bond strengths obtained revealed that the adhesive failure occurred at a lower force than the C2 cohesive failure, irrespective of the cement type or the surface treatment applied (p-value M-W<0.001).

Furthermore, both for cohesive and adhesive failures, dual-polymerizing cements presented higher bond strength values than autopolymerizing cements.

-Electron microscopy

**Figure 2:**
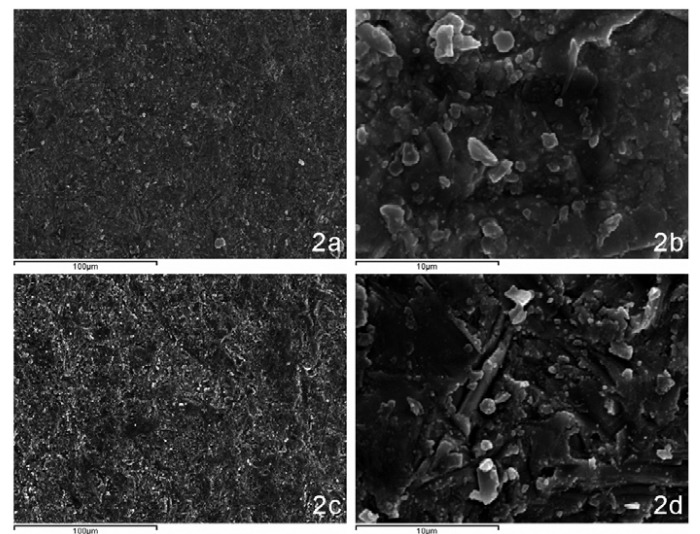
a) Lava 500x SEM. b) Lava 5000x SEM. c) Lava sandblasted 500x SEM. d) Lava sandblasted 5000x SEM.

**Figure 3:**
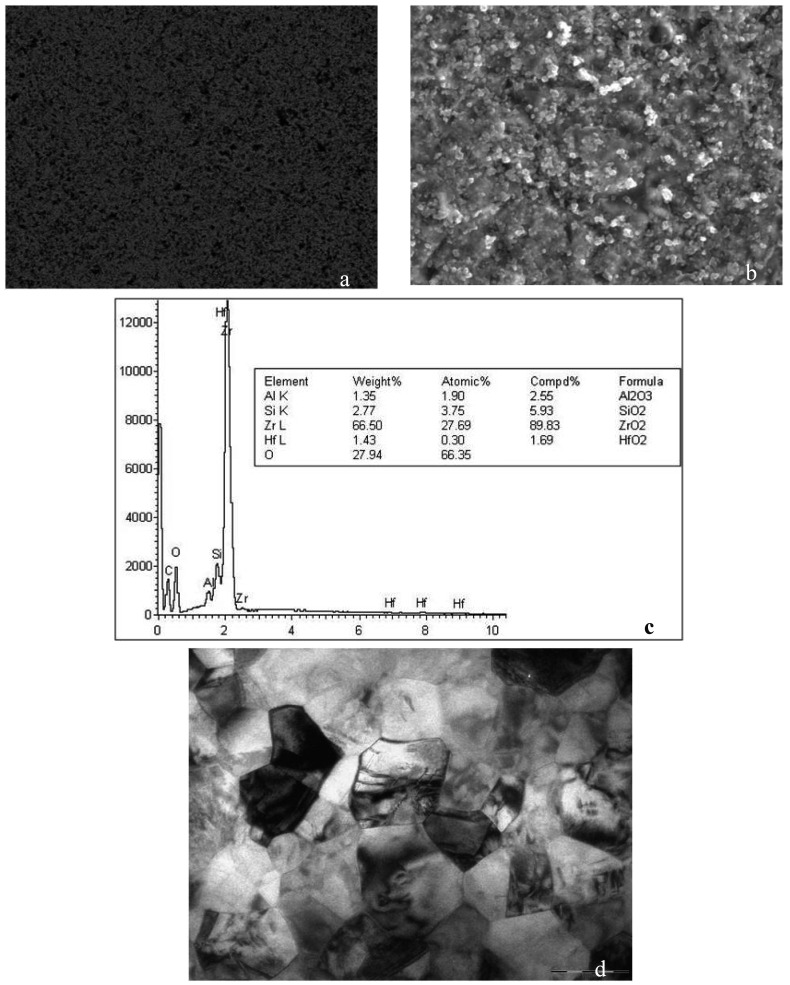
a) Lava silica coated 500x BSE. b) Lava silica coated 5000x SEM. c) Composition analysis of the silica-coated Lava ceramic. d) Polygonal crystals with hexagonal appearance, variable in size but averaging around 0.5 micrometres (8900x TEM).

SEM examination of the Lava TM ceramic revealed a smooth, polished, homogenous surface with a regular pattern due to the CAD-CAM method of manufacture (Fig.2). After sandblasting, the surfaces were more irregular in shape and relief, having a disorderly pattern with a multitude of particle fragments with a varied morphology (Fig. 2). The silica-coated ceramics presented a less pronounced irregularity, better appreciated at higher magnifications (Fig. 3). The composition analysis confirmed the presence of silica in the ceramic surface, which was not present in the analysis of the sample without surface treatment (Fig. 3).

TEM of Lava TM revealed a structure of polygonal crystals about 0.5 micrometers in size, with no interstices, glassy matrix or pores between them (Fig. 3).

## Discussion

The shear test results indicated the best values for mechanical and chemical treatment combinations for each cement (although without significant differences between them) ( Table 2).

• Panavia F: Lava + sandblasting + Clearfil SE Bond Primer + Porcelain Activator + Panavia F = 14.9±5.01 MPa. (Group 4) and Lava + CoJet + Silane + Clearfil Se Bond Primer + Panavia F 11.66 ± 2.83 MPa (Group 5).

• Variolink II: Lava + CoJet Sand + Clearfil Ceramic Primer + Variolink II = 13.44±3.53 MPa. (Group 3), Lava + CoJet + Silane + Variolink II 10.49 ± 2.82 MPa (Group 2) and Lava + sandblasting + silane + Variolink II 10.58 ± 4.44 MPa (Group 1).

• Rely X: Lava + CoJet Sand + Espe-sil + Rely X = 10.83±4.77 MPa. (Group 8)

• Multilink: Lava + CoJet Sand + Clearfil Ceramic primer + Multilink = 8.96 ± 3.27 MPa. (Group 13).

Most authors (1,4,13,14,18) agree on the effectiveness of Panavia F, and that this success is due to the MDP monomer. Nevertheless, there is some disagreement between authors regarding the Bis-GMA resin cements, such as Variolink II o Multilink. Some authors (4,8) cite the importance of MDP because of the chemical reaction with the ceramic, while the Bis-BMA based cements will have a mainly micromechanical retention. In addition, they explain that thermocycling reduces the adhesion values of cements with Bis-GMA, whereas cements with MDP are not altered. Our study found no significant differences between the dual polymerizing cements (Variolink II and Panavia F), although there were differences between these and the autopolymerizing cements (Rely X and Multilink).

Likewise, dual-polymerizing cements performed better than the autopolymerizing, with statistically significant differences (p-value M-W<0.001).

Nevertheless, since the bonding capacity of each cement may differ when cemented to the tooth, the choice of cement cannot be based on bond strengths alone. A similar cross study, examining bonding with teeth, and combined with our data would give a more objective view of the ideal choice in each case. On the other hand, given the proximity to statistical significance (acceptance threshold p=0.001) for the comparison between some combinations, it is quite probable that a further study with a larger sample size would statistically confirm some of the descriptive trends already observed between the seven groups with the highest values. Further studies with a larger sample size subjected to thermocycling are necessary in order to find the best combination for ceramic bonding.

With regard to surface treatment, the best combination incorporated sandblasting (G4), with no statistically significant differences with the silica-coated group (G3). Sandblasting increases the irregularity of the zirconium surface, improving the interdigitation with the cement. Silica coating not only generates a roughened surface, but also incorporates silica particles into the ceramic. The presence of silica (SiO2) and free hydroxyl groups allows the formation of siloxane bridges between the ceramic and acrylic resin groups (9,10). The surface treatments influence and optimize the bond strengths, as stated by other authors (1,7,9,14-16).

The use of Clearfil, whether a one-bottle (Ceramic Primer (MDP resin and silane) or two-bottle system (SE Bond Primer (resin with MDP) and Porcelain Activator (silane), improves the bond strengths for aluminum oxide ceramic (p-value M-W<0.001) (2,14). The use of a monomer containing silane and MDP combined with silica coating was studied with respect to the bond strengths of the four cements [9]; however, as with surface treatments, the best possible combination is not always obtained. We also studied whether the use of one or two-bottle presentations had any influence (groups 5 and 6), finding no significant differences.

Electron microscopy observation of the zirconium samples, both before and after sandblasting or silica coating, revealed changes in the ceramic, from a smooth, homogeneous surface and equiaxial polygonal structure to an unstructured and irregular surface.

Several authors have observed that a silica-coated zirconium oxide surface has a finer surface roughness than a surface sandblasted with aluminum oxide (9,12,16), which by electron microscopy appears to have a rougher surface due to the presence of micro-retentive grooves (2,11,16).

Once the ceramic is silica coated, silicon is detected in the composition analysis, indicating that CoJet Sand particles have been incorporated into the ceramic surface (12,19), explaining why generally better bond strengths are obtained for resin cements (Variolink II, Rely X and Multilink) after silica coating (2,12,20).

## Conclusions

Within the limits of the present study, it was observed that:

1. Sandblasting and silica coating modify the surface of the zirconium-oxide ceramic, creating a rougher more retentive surface, and providing a better mechanical interlocking with the ceramic and the cement.

2. The composition analysis demonstrates that the abrasive CoJet Sand particles contain silicon; this becomes part of the Lava ceramic following surface treatment, possibly influencing the improvement in bond strengths.

3. In this study, the better bond strengths were obtained by dual-polymerizing cements associated with sandblasting or silica coating and an adhesive containing MDP.

4. Adhesive failure (separation of the cement and ceramic) occurs at a lower force than cohesive failure (fracture of cement).

5. For autopolymerizing cements, silica coating the ceramic is preferable to.
